# Prognostic Impact of Adjuvant Immunotherapy in Patients With Resectable NSCLC After Neoadjuvant Chemoimmunotherapy: A Brief Report

**DOI:** 10.1016/j.jtocrr.2024.100763

**Published:** 2024-11-12

**Authors:** Yichen Dong, Long Xu, Jialiang Wen, Haojie Si, Juemin Yu, Tao Chen, Huikang Xie, Xinjian Li, Minglei Yang, Junqiang Fan, Junqi Wu, Yunlang She, Deping Zhao, Chang Chen

**Affiliations:** a Department of Thoracic Surgery, Shanghai Pulmonary Hospital, Tongji University School of Medicine, Shanghai, People's Republic of China; b Department of Pathology, Shanghai Pulmonary Hospital, Tongji University School of Medicine, Shanghai, People's Republic of China; c Department of Thoracic Surgery, Ningbo Hospital, Zhejiang University, Ningbo, People's Republic of China; d Department of Thoracic Surgery, Ningbo Number 2 Hospital, Chinese Academy of Sciences, Zhejiang, People's Republic of China; e Department of Thoracic Surgery, the Second Affiliated Hospital of Zhejiang University School of Medicine, Hangzhou, People's Republic of China

**Keywords:** Non-small cell lung cancer, Neoadjuvant chemoimmunotherapy, Adjuvant immunotherapy, Perioperative immunotherapy

## Abstract

**Objective:**

The potential survival benefits of adjuvant immunotherapy for resectable NSCLC after neoadjuvant chemoimmunotherapy, and the optimal number of adjuvant immunotherapy cycles, remain uncertain. This study aims to evaluate the prognostic impact of adjuvant immunotherapy and determine the optimal number of cycles.

**Methods:**

A total of 438 patients who received neoadjuvant chemoimmunotherapy between August 2019 and June 2022 across four hospitals were enrolled in this study, with a median follow-up time of 31.3 months. Recurrence-free survival (RFS) and overall survival (OS) were estimated using Kaplan-Meier methods and tested by log-rank test. Unstratified Cox proportional hazards models were fitted to the subgroups.

**Results:**

In this multi-center cohort, 29.7% of patients (n = 130) achieved a pathologic complete response. Patients who received adjuvant immunotherapy experienced significant survival benefits compared with those who did not (RFS: hazard ratio [HR] = 0.63, 95% confidence interval: 0.41–0.98, *p* = 0.037; OS: hazard ratio = 0.27, 95% confidence interval: 0.13–0.57, *p* < 0.001). Subgroup analyses found that patients with a squamous histologic type, positive PD-L1 expression, and those with a major pathologic response particularly benefited from adjuvant immunotherapy. In addition, we found that six cycles of adjuvant immunotherapy served as a threshold for better prognostic differentiation, suggesting that six or more cycles may be more beneficial.

**Conclusions:**

Our study found that the addition of adjuvant immunotherapy to neoadjuvant chemoimmunotherapy is significantly associated with improved RFS and OS in patients with resectable NSCLC. We also identified that six cycles of adjuvant immunotherapy may be the optimal regimen for these patients.

## Introduction

In recent years, several neoadjuvant immunotherapy trials^1^, ^2^, ^3^, ^4^, ^5^ have revolutionized the treatment paradigm for early-stage NSCLC. Neoadjuvant immunotherapy has increased the rate of pathologic complete response (pCR) to 17.2% to 24.8% and extended the median event-free survival (EFS) to over 31.6 months compared with chemotherapy.^1^, ^2^, ^3^, ^4^, ^5^ These clinical trials featured different study designs: CheckMate816 tested only the neoadjuvant approach, whereas AEGEAN, Neotorch, Checkmate77T, and KEYNOTE-671 incorporated additional adjuvant immunotherapy.

An indirect meta-analysis of these five clinical trials suggests that perioperative immunotherapy does not provide a significant EFS benefit over neoadjuvant immunotherapy alone, but it does result in significantly higher rates of treatment-related adverse events.^6^ Nevertheless, the CheckMate-77T study indicated that for patients who achieved a pCR, postoperative adjuvant immunotherapy significantly improved EFS compared with chemotherapy (hazard ratio [HR] = 0.21, 95% confidence interval [CI]: 0.04–1.04). Conversely, for patients who did not achieve pCR, continuing adjuvant nivolumab treatment offered limited EFS benefit compared with chemotherapy (HR = 0.70, 95% CI: 0.43–1.13). This suggests that adjuvant immunotherapy may benefit specific patient subgroups.

In the adjuvant phase of perioperative immunotherapy clinical trials, patients typically receive adjuvant immunotherapy for one year.^7^ Nevertheless, only 26.3% to 43.6% of patients complete the full year of adjuvant immunotherapy. In the NADIM II trial, patients were randomized to receive neoadjuvant nivolumab plus chemotherapy followed by adjuvant nivolumab for 6 months in the experimental arm, with 66.0% completing the scheduled adjuvant immunotherapy program.^8^ Nevertheless, the difference in adjuvant treatment duration did not translate into a significant difference in prognosis. A summary of the details of these clinical trials can be found in Supplementary Table 1.

In this study, we aimed to explore the necessity of adjuvant immunotherapy after neoadjuvant immunotherapy and to determine the optimal number of adjuvant immunotherapy cycles.

## Material and Methods

### Patients and Study Design

This retrospective study was approved by the Ethical Review Board of Shanghai Pulmonary Hospital (Institutional Review Board number: L24-386), and consents was waived due to the retrospective design. Patients diagnosed with clinical stage IB to IIIB NSCLC (according to the American Joint Committee on Cancer Staging Manual, Eighth Edition), with *EGFR* and *ALK*-negative tumors, who received neoadjuvant chemoimmunotherapy between August 2019 and June 2022 at Shanghai Pulmonary Hospital, the Second Affiliated Hospital of Zhejiang University School of Medicine, Ningbo Hospital of Zhejiang University, and Ningbo Number 2 Hospital were enrolled. Adjuvant treatment decisions were evaluated by a multidisciplinary team comprising surgeons, medical oncologists, pathologists, and radiologists. This team developed treatment plans on the basis of each patient's pathological response, pathological lymph node staging, PD-L1 status, personal preferences, and Eastern Cooperative Oncology Group performance-status score.

### Pathological Assessment

Pathological evaluation of all patients was performed by two senior pathologists following the International Association for the Study of Lung Cancer recommended guidelines.^9^ Pathologic complete response was defined as the absence of viable tumor cells in both the primary tumor bed and sampled lymph nodes. Major pathologic response (MPR) was defined as having no more than 10% viable tumor cells in the primary tumor bed.^9^

### Follow-Up Strategy

Overall survival (OS) was measured from the date of surgery to death for any cause. Recurrence-free survival (RFS) was defined as the time from surgery to the recurrence of the disease. All patients in this study were followed up until May 20, 2024, with a median follow-up duration of 31.3 months.

### Statistical Analysis

Recurrence-free survival and OS were estimated using the Kaplan-Meier method and compared across groups using the log-rank test. Unstratified Cox proportional hazards models were fitted to the subgroups. Propensity-score matching (PSM) was employed to minimize the influence of potential prognostic confounders. The optimal cut-off value of adjuvant immunotherapy cycles was determined using maximally selected rank statistics. All statistical analyses were performed using R software (version 4.3.1), and a two-sided *p* value of less than 0.05 was considered statistically significant.

## Results

### Patient Characteristics

A total of 438 patients were finally included in the analytic cohort, with a consort diagram presented in Supplementary Figure 1. Among them, 29.68% of patients (n = 130) reached pCR. A total of 176 patients received adjuvant immunotherapy after surgery, with a median duration of 11 cycles. In addition, patients receiving adjuvant immunotherapy were younger compared with those who did not receive this treatment (Supplementary Table 2).

### Prognostic Value of Adjuvant Immunotherapy

**Figure 1:**
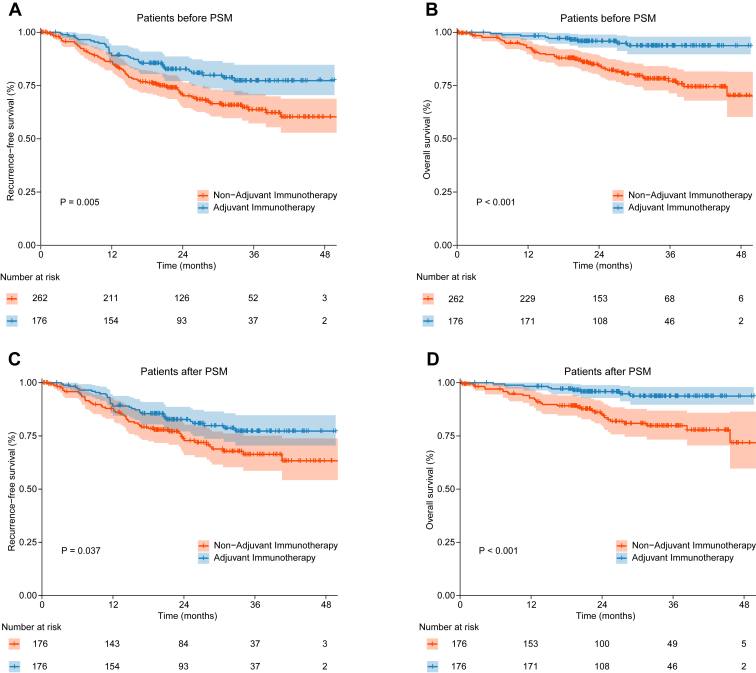
Prognostic differences with and without adjuvant immunotherapy. *(A)* RFS on the basis of whether adjuvant immunotherapy was administered in patients before PSM. *(B)* OS on the basis of whether adjuvant immunotherapy was administered in patients before PSM. *(C)* RFS on the basis of whether adjuvant immunotherapy was administered in patients after PSM. *(D)* OS on the basis of whether adjuvant immunotherapy was administered in patients after PSM. OS, overall survival; PSM, propensity-score matching; RFS, recurrence-free survival.

Patients who received adjuvant immunotherapy had significantly better RFS and OS compared with those who did not receive adjuvant immunotherapy (RFS: HR = 0.57, 95% CI: 0.38–0.84, *p* = 0.005, Fig. 1*A*; OS: HR = 0.25, 95% CI: 0.12–0.50, *p* < 0.001, Fig. 1*B*). To minimize the influence of potential prognostic confounders, PSM was used to match patients in the adjuvant immunotherapy group with those in the non-adjuvant immunotherapy group on the basis of age, sex, smoking history, pathological N-stage, pathologic response of primary tumor, histological type, and PD-L1 expression, at a one-to-one ratio. The demographic and clinical variables were well-balanced (Table 1).

**Table 1:** Baseline Characteristics of Patients Receiving Adjuvant Immunotherapy Compared to Those Not After PSM

	All Patients	Adjuvant Immunotherapy	Observation	*p*
No. of patients	352	176	176	
Age, y, n (%)				1.000
<65	209 (59.4)	105 (59.7)	104 (59.1)	
≥65	143 (40.6)	71 (40.3)	72 (40.9)	
Sex, n (%)				0.325
Female	28 (7.95)	17 (9.66)	11 (6.25)	
Male	324 (92.0)	159 (90.3)	165 (93.8)	
Smoking history, n (%)				0.658
Never smoked	129 (36.6)	67 (38.1)	62 (35.2)	
Current or former smoker	223 (63.4)	109 (61.9)	114 (64.8)	
PD-L1, n (%)				0.925
0%	176 (50.0)	86 (48.9)	90 (51.1)	
1%–49%	63 (17.9)	33 (18.8)	30 (17.0)	
≥50%	53 (15.1)	28 (15.9)	25 (14.2)	
NA	60 (17.0)	29 (16.5)	31 (17.6)	
Neoadjuvant cycles, n (%)				0.225
2–3	281 (79.8)	134 (76.1)	147 (83.5)	
4	59 (16.8)	35 (19.9)	24 (13.6)	
>4	12 (3.41)	7 (3.98)	5 (2.84)	
Histological type, n (%)				0.821
Non-Sqcc	117 (33.2)	57 (32.4)	60 (34.1)	
Sqcc	235 (66.8)	119 (67.6)	116 (65.9)	
Pathologic response (PT)				0.517
Non-MPR	147 (41.8)	70 (39.8)	77 (43.8)	
MPR	205 (58.2)	106 (60.2)	99 (56.2)	
Pathologic N stage				0.882
N0	233 (66.2)	117 (66.5)	116 (65.9)	
N1	47 (13.4)	22 (12.5)	25 (14.2)	
N2	72 (20.5)	37 (21.0)	35 (19.9)	

MPR, major pathologic response; PD-L1, programmed cell death-ligand 1; PT, primary tumor; PSM, propensity-score matching; Sqcc, squamous cell carcinoma.

After PSM, patients who received adjuvant immunotherapy continued to experience significantly improved RFS and OS compared with those who did not receive the treatment (RFS: HR = 0.63, 95% CI: 0.41–0.98, *p* = 0.037, Fig. 1*C*; OS: HR = 0.27, 95% CI: 0.13–0.57, *p* < 0.001, Fig. 1*D*). In the MPR subgroup, patients receiving adjuvant immunotherapy had significantly better prognosis than those who did not undergo adjuvant immunotherapy (RFS: HR = 0.41, 95% CI: 0.20–0.81, *p* = 0.008; OS: HR = 0.12, 95% CI: 0.03–0.54, *p* < 0.001) (Supplementary Fig. 2*A* and *B*). Nevertheless, no significant difference was achieved in the non-MPR subgroup between those who received adjuvant immunotherapy and those who did not (RFS: HR = 0.92, 95% CI: 0.52–1.63, *p* = 0.770; OS: HR = 0.44, 95% CI: 0.18–1.06, *p* = 0.058) (Supplementary Fig. 2*C* and *D*). Further subgroup analyses using a forest plot indicated that patients with a squamous histologic type benefitted more from adjuvant immunotherapy compared with those with non-squamous histology. In addition, patients with positive PD-L1 expression tended to derive more favorable outcomes from adjuvant immunotherapy (Supplementary Fig. 3*A* and *B*).

### Appropriate Number of Cycles of Adjuvant Immunotherapy

**Figure 2:**
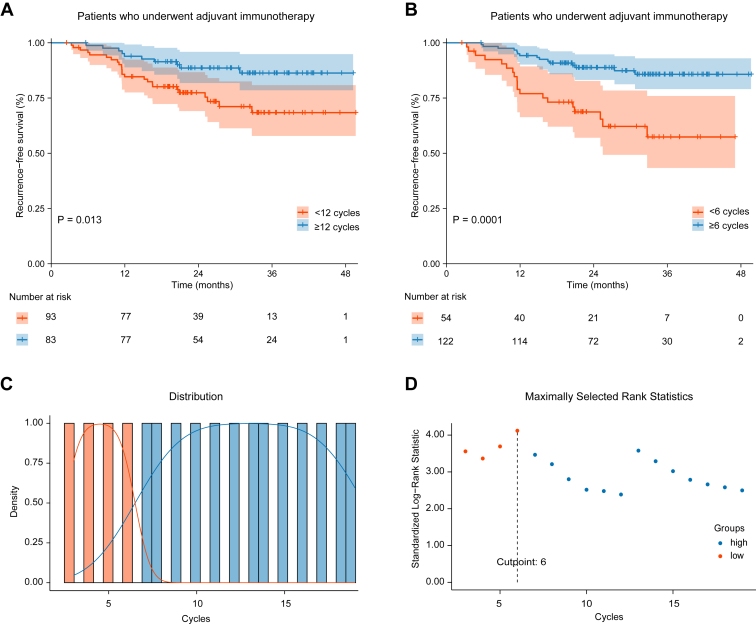
Optimal number of cycles of adjuvant immunotherapy. *(A)* RFS in patients who underwent adjuvant immunotherapy using 12 cycles as the adjuvant immunotherapy cycle boundary *(B)* RFS in patients who underwent adjuvant immunotherapy using six cycles as the adjuvant immunotherapy cycle boundary. *(C)* The distribution of cycles for adjuvant immunotherapy *(D)* Analysis of cut-off values for the number of cycles of adjuvant immunotherapy. RFS, recurrence-free survival.

First, we compared the prognosis for patients receiving greater than or equal to 12 cycles of adjuvant immunotherapy, the typical number used in clinical trials,^7^ to those receiving fewer than 12 cycles. The results reported that extending adjuvant immunotherapy beyond one year provided additional survival benefits compared with treatment lasting less than one year (RFS: HR = 0.40, 95% CI: 0.19–0.84, *p* = 0.013, Fig. 2*A*). Nevertheless, only 47.2% of patients completed a full year of treatment in the adjuvant immunotherapy cohort.

The distribution of adjuvant immunotherapy cycles is shown in Figure 2*C*, and the cut-off value analysis (Fig. 2*D*) identified six cycles as the optimal number, with the standardized log-rank statistic reaching its maximum value of 4.12. Patients who received more than six cycles had significantly better RFS compared with those who received fewer than six cycles (RFS: HR = 0.29, 95% CI: 0.15–0.57, *p* = 0.001, Fig. 2*B*). Nevertheless, this extended treatment resulted in a higher incidence of treatment-related adverse events of grade 3 to 5 (Supplementary Table 3). Supplementary Figure 4 reported the cut-off analysis for adjuvant immunotherapy at cycles 3, 9, and 18, with HRs of 0.33, 0.33, and 0.32, respectively. The HR value for six cycles was significantly lower than that for other cycles. Moreover, up to 69.3% of patients completed six cycles of treatment in the adjuvant immunotherapy cohort. These data suggest that six cycles of adjuvant immunotherapy may be a more practical and effective option, though further validation in clinical trials is necessary.

## Discussion

The efficacy of neoadjuvant immunotherapy has been reported in previous phase III clinical trials.^1^^,^^2^^,^^8^ Not only is neoadjuvant immunotherapy effective, but studies such as IMpower-010^10^ and PEARLS^11^ have highlighted the essential role of adjuvant immunotherapy in early-stage NSCLC. Nevertheless, KEYNOTE-091 reported that adjuvant immunotherapy did not improve disease-free survival in the 50% and higher PD-L1 subgroup. We found that PD-L1 expression influenced the extent of benefit from postoperative adjuvant immunotherapy after neoadjuvant immunotherapy. In Addition, the results of the pathological evaluation could guide postoperative adjuvant treatment strategies. Specifically, patients who achieved MPR and had a squamous histologic type benefited significantly from adjuvant immunotherapy. This suggests a need for better regimens for patients who did not achieve MPR, as they are likely resistant to immunotherapy.

Currently, there is no consensus on the optimal number of cycles for adjuvant immunotherapy. A one-year cycle of adjuvant immunotherapy is most often recommended in clinical trials and practice. Nevertheless, in our adjuvant immunotherapy cohort, only 47.2% of patients completed a full year of treatment. We found that six cycles of adjuvant immunotherapy might be a better option, effectively balancing tumor eradication with quality of life and being easier to implement. Nevertheless, this approach still requires further validation in subsequent clinical trials.

Our study has several limitations. First, our treatment effectiveness estimates might be confounded by the patient selection bias owing to the retrospective nature of the study. In addition, the number of patients who received adjuvant immunotherapy is not sufficiently large. Hence, large-scale clinical trials are warranted in the future.

## Conclusions

To our knowledge, this is the first study to report that the addition of adjuvant immunotherapy to neoadjuvant chemoimmunotherapy is significantly associated with improved RFS and OS in patients with resectable NSCLC. In addition, we found that six cycles of adjuvant immunotherapy may be a more effective option for patients who have received neoadjuvant chemoimmunotherapy.

## CRediT Authorship Contribution Statement

**Yichen Dong:** Conceptualization, Methodology, Formal analysis, Investigation, Data curation, Writing - original draft, Visualization.

**Long Xu:** Conceptualization, Methodology, Formal analysis, Investigation, Data curation, Writing - review & editing, Visualization.

**Jialiang Wen:** Conceptualization, Methodology, Formal analysis, Investigation, Data curation, Writing - review & editing, Visualization.

**Haojie Si:** Investigation, Data curation.

**Juemin Yu:** Investigation, Data curation.

**Tao Chen:** Investigation, Data curation.

**Huikang Xie:** Investigation, Data curation.

**Xinjian Li:** Investigation, Data curation.

**Minglei Yang:** Investigation, Data curation.

**Junqiang Fan:** Investigation, Data curation.

**Deping Zhao:** Investigation, Data curation.

**Junqi Wu:** Methodology, Resource, Data curation, Investigation.

**Yunlang She:** Methodology, Resource, Data curation, Investigation.

**Chang Chen:** Conceptualization, Investigation, Writing - review & editing, Visualization, Supervision, Project administration.

## Disclosure

The authors declare no conflict of interest.
