# Supplementary figures and images for: Prognostic Impact of Adjuvant Immunotherapy in Patients With Resectable NSCLC After Neoadjuvant Chemoimmunotherapy: A Brief Report
Source: JTO Clin Res Rep. 2024 Nov 12;6(1):100763. doi: 10.1016/j.jtocrr.2024.100763 (PMC11699361; doi:10.1016/j.jtocrr.2024.100763)

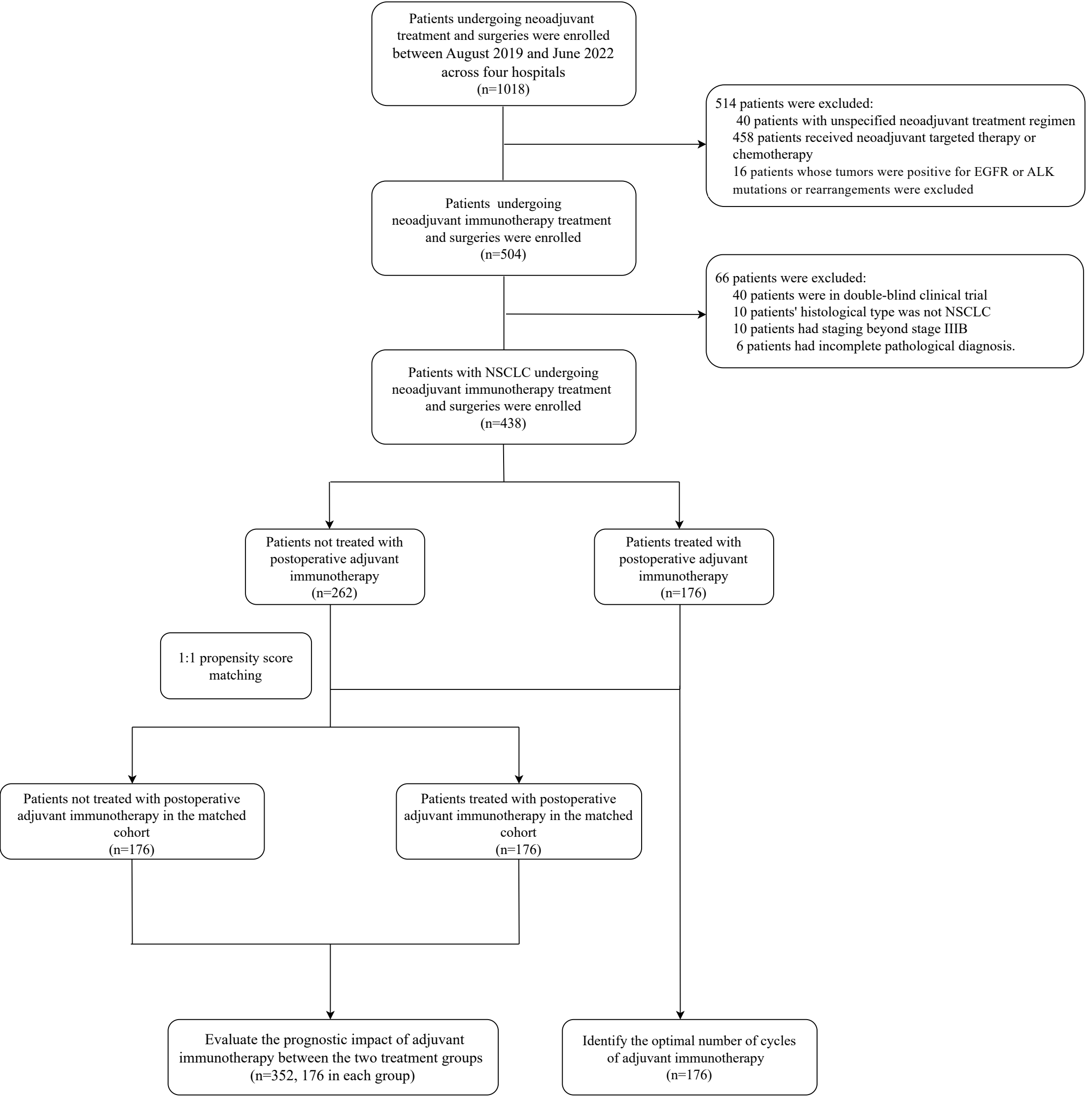

Supplement: Supplementary Figure 1 [file mmc1.pdf]

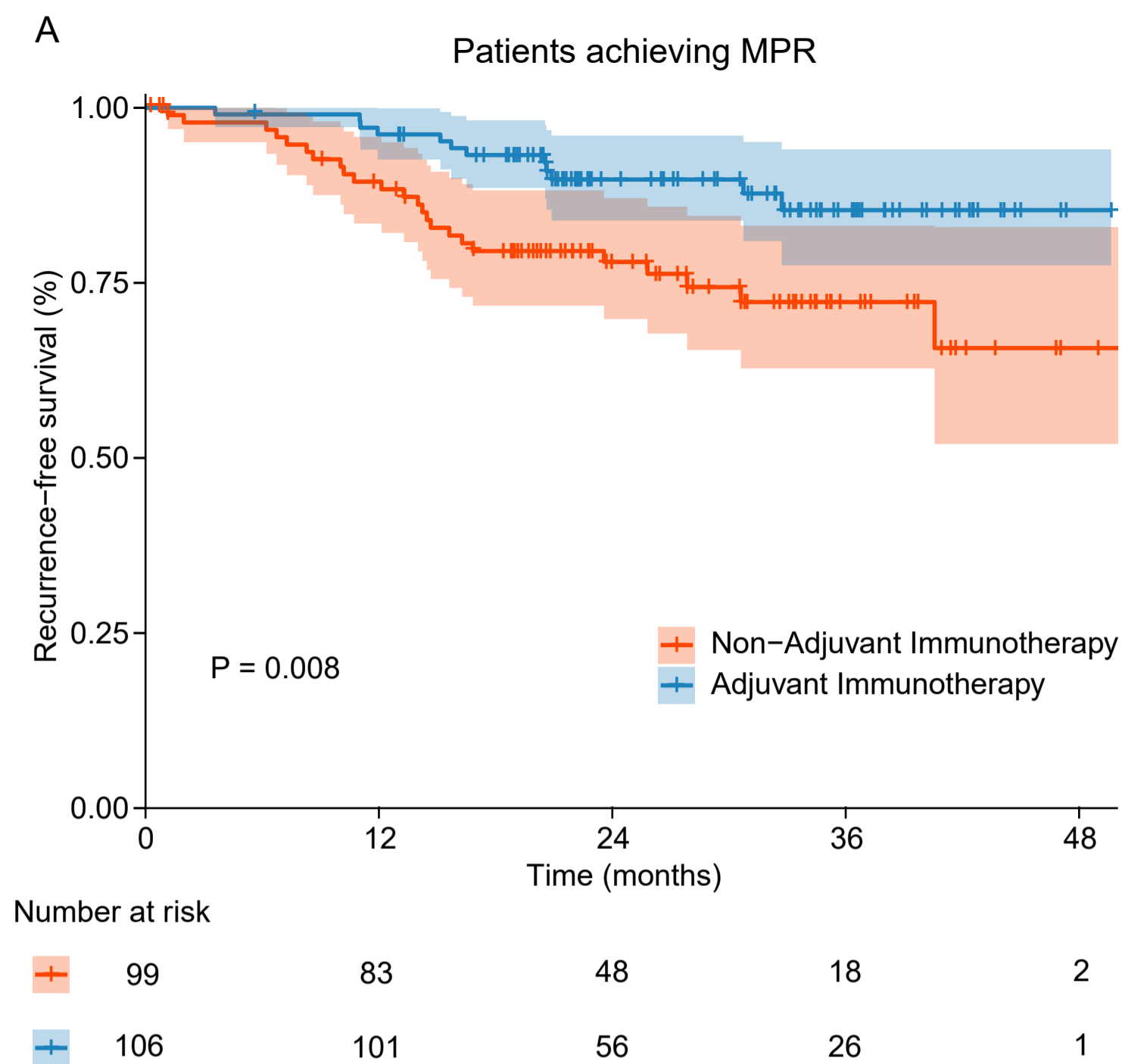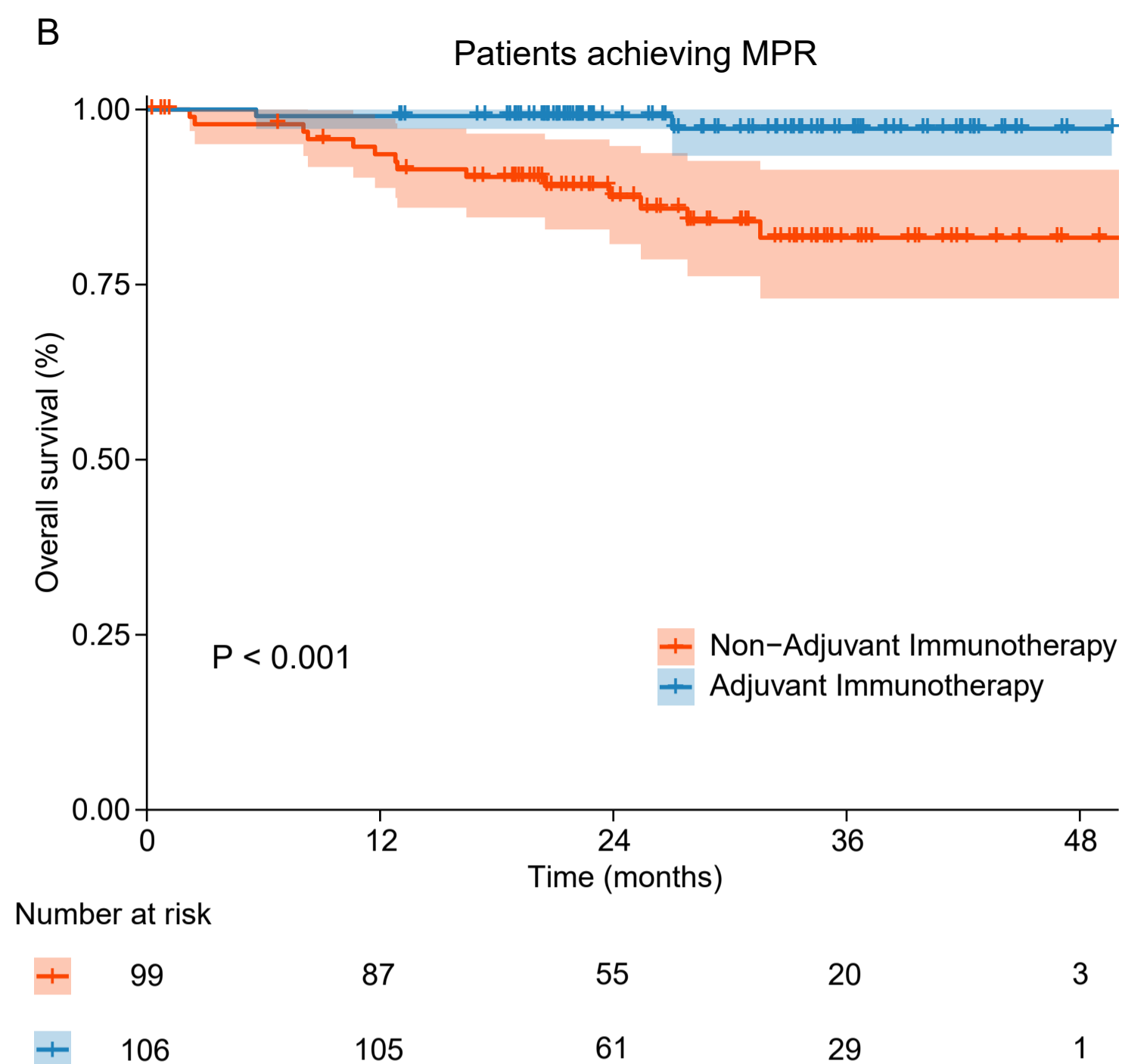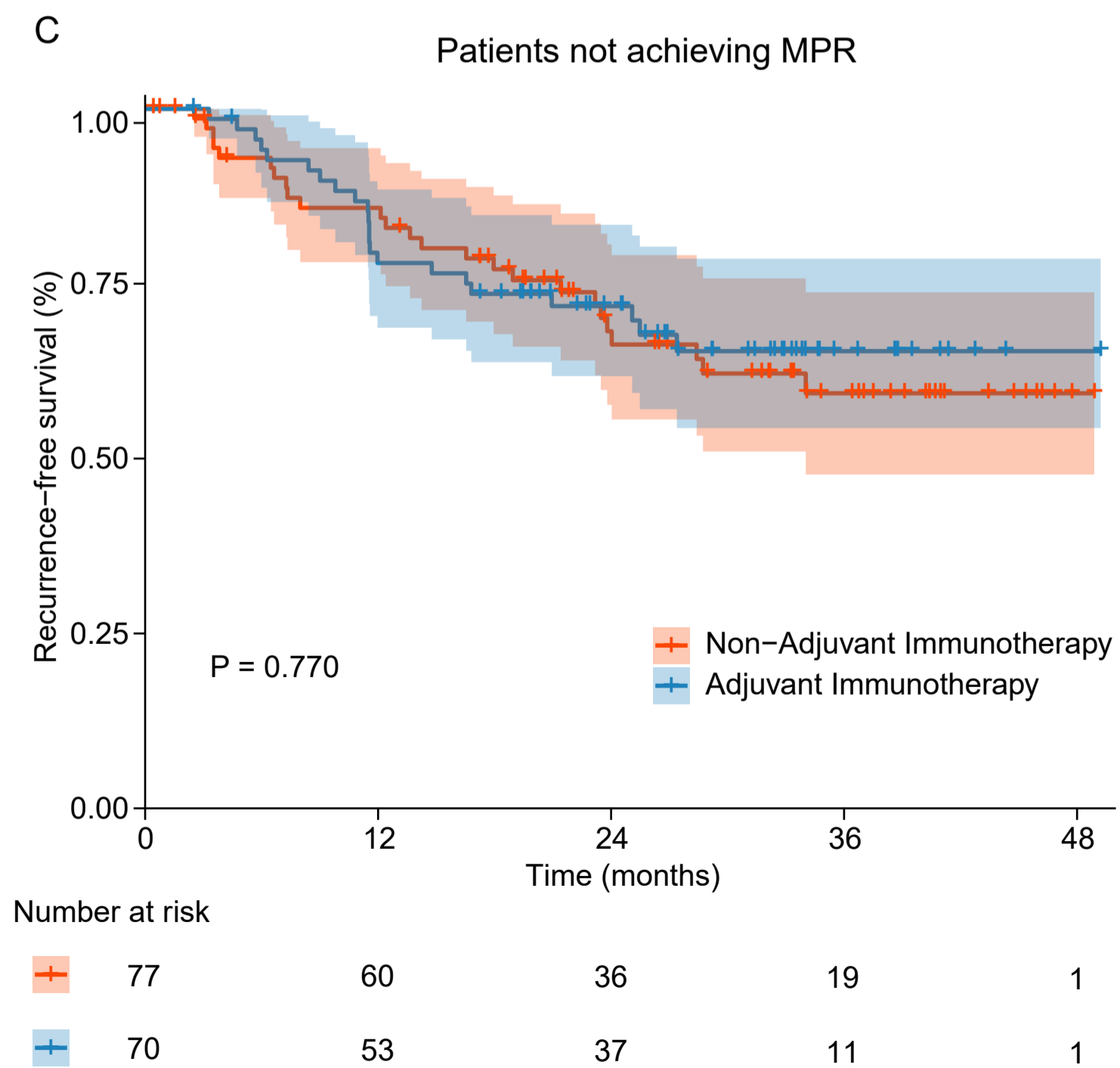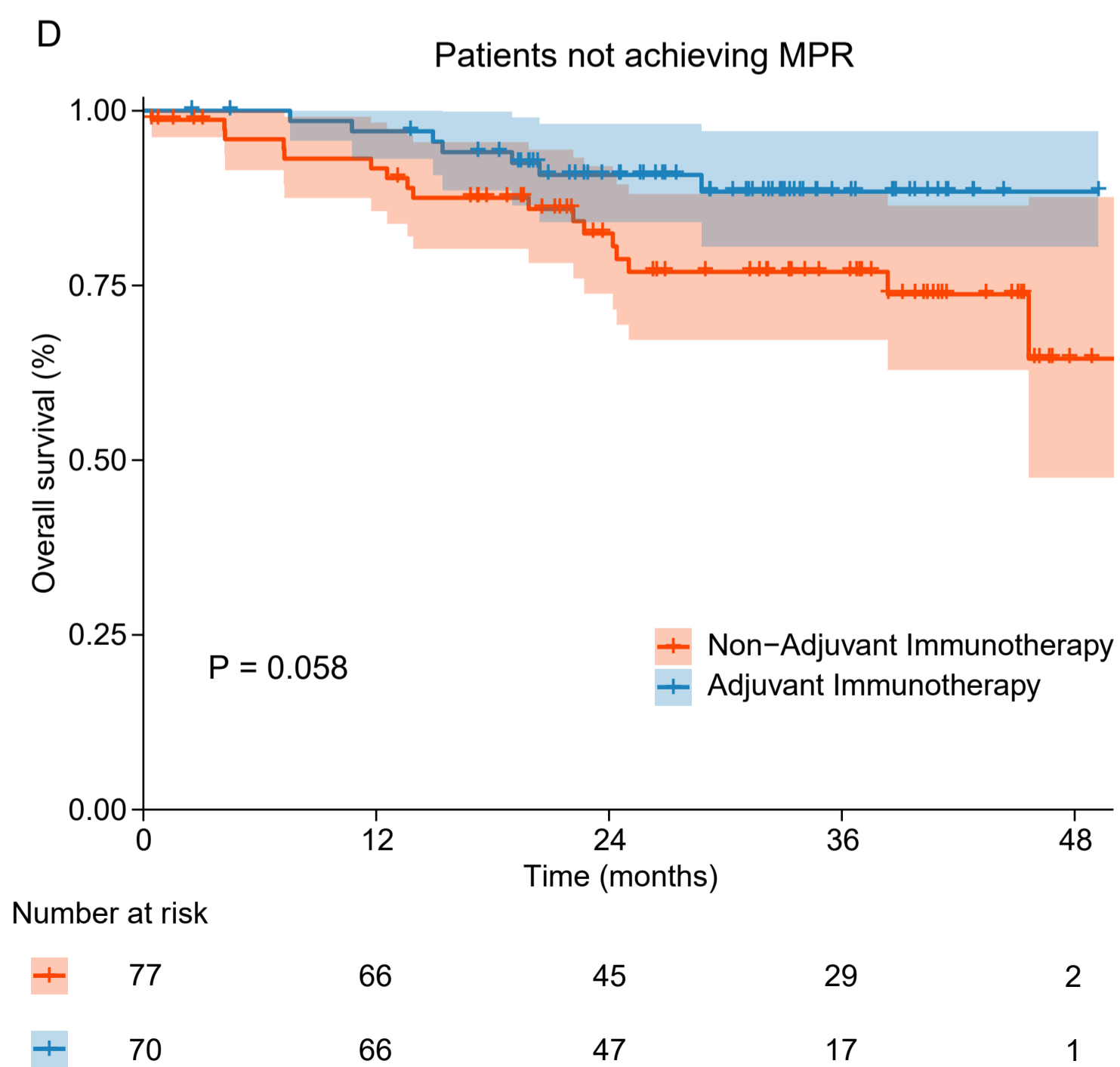

Supplement: Supplementary Figure 2 [file mmc2.pdf]

A

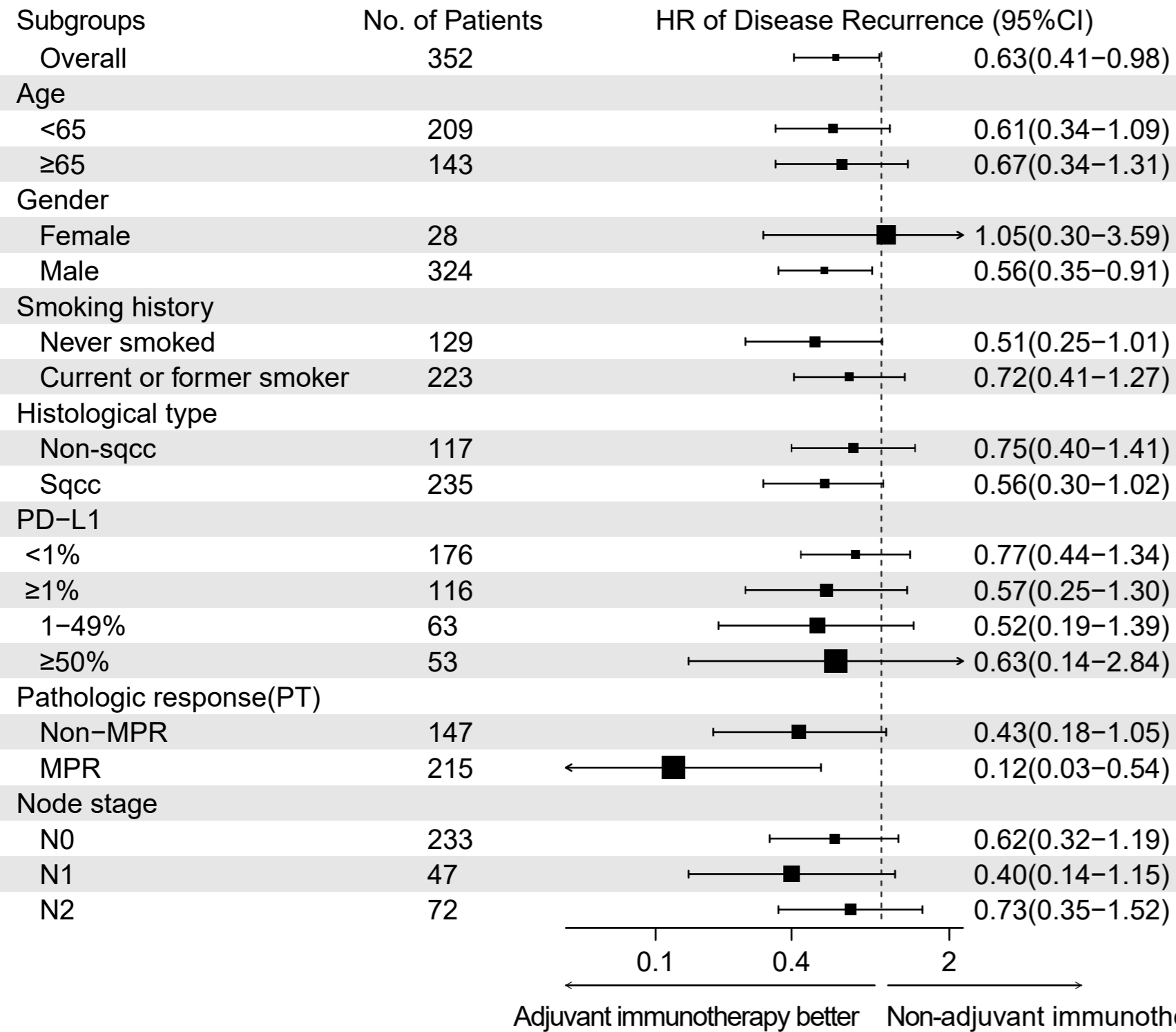

B

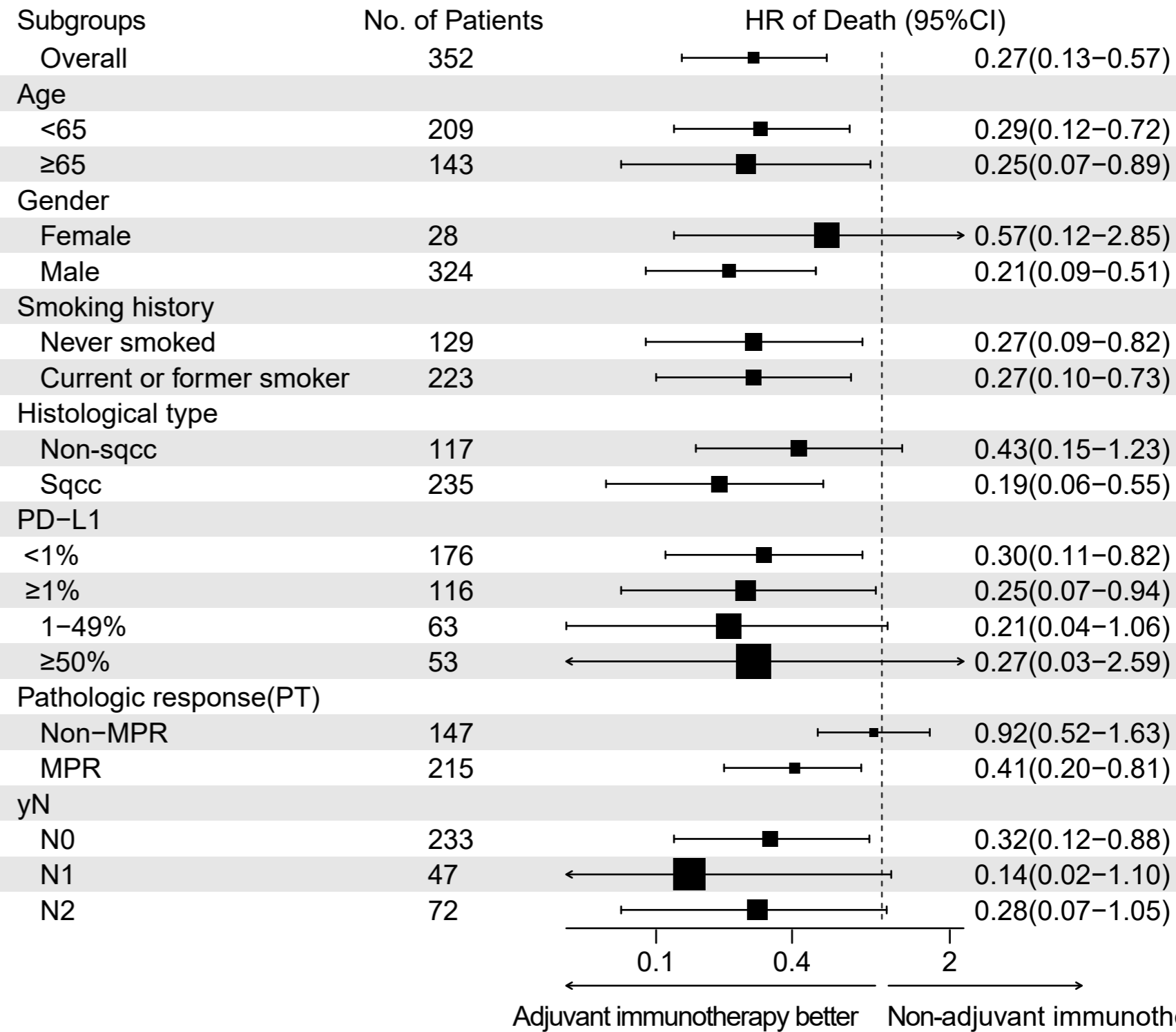

Supplement: Supplementary Figure 3 [file mmc3.pdf]
