# Supplementary material for: Prognostic Impact of Adjuvant Immunotherapy in Patients With Resectable NSCLC After Neoadjuvant Chemoimmunotherapy: A Brief Report
Source: JTO Clin Res Rep. 2024 Nov 12;6(1):100763. doi: 10.1016/j.jtocrr.2024.100763 (PMC11699361; doi:10.1016/j.jtocrr.2024.100763)
Supplement: Supplementary Figure 4 [file mmc4.pdf]

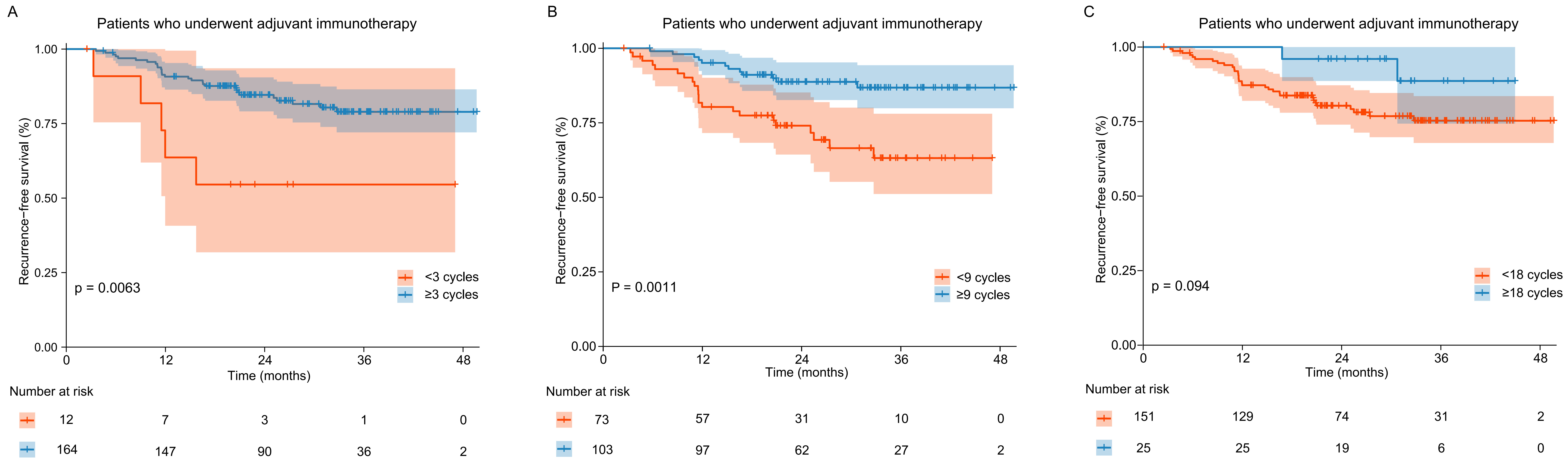
A
Patients who underwent adjuvant immunotherapy
Recurrence−free survival (%)
1.00
0.75
0.50
0.25
0.00
0
12
24
36
48
Time (months)
p = 0.0063
<3 cycles
≥3 cycles
Number at risk
12 7 3 1 0
164 147 90 36 2
B
Patients who underwent adjuvant immunotherapy
Recurrence−free survival (%)
1.00
0.75
0.50
0.25
0.00
0
12
24
36
48
Time (months)
P = 0.0011
<9 cycles
≥9 cycles
Number at risk
73 57 31 10 0
103 97 62 27 2
C
Patients who underwent adjuvant immunotherapy
Recurrence−free survival (%)
1.00
0.75
0.50
0.25
0.00
0
12
24
36
48
Time (months)
p = 0.094
<18 cycles
≥18 cycles
Number at risk
151 129 74 31 2
25 25 19 6 0
