# Supplementary material for: Prognostic Impact of Adjuvant Immunotherapy in Patients With Resectable NSCLC After Neoadjuvant Chemoimmunotherapy: A Brief Report
Source: JTO Clin Res Rep. 2024 Nov 12;6(1):100763. doi: 10.1016/j.jtocrr.2024.100763 (PMC11699361; doi:10.1016/j.jtocrr.2024.100763)
Supplement: Supplementary Table 1 [file mmc5.docx]

**Supplementary table 1. A Summary of Neoadjuvant Immunotherapy Clinical Trials**

| Trial name | Trial design | Therapeutic plan | Target Stage | MPR rate | pCR rate | 2-years  EFS | Median EFS & HR | Median OS & HR | Adjuvant immunotherapy completion rate |
| --- | --- | --- | --- | --- | --- | --- | --- | --- | --- |
| CheckMate 816 | Neoadjuvant, phase III | Cycle: 3 (Q3W)  Nivo+CTx→R | IB(>4cm)-IIIA (AJCC v7) | 36.9% vs. 8.9% | 24.0% vs. 2.2% | 63.8% vs. 45.3% | HR, 0.63  (31.6 vs. 20.8m) | HR, 0.57  NR | NA |
| NADIM II | Neoadjuvant+adjuvant  , phase II | Cycle: 3 (Q3W) +6(Q4W)  Nivo+CTx→R→Nivo | IIIA-IIIB (AJCC v8) | 52.6% vs. 13.8% | 36.8% vs. 6.9% | 67.2% vs. 40.9% | HR, 0.47  (NR vs. 15.4m) | HR, 0.43  NR | 66.0% |
| CheckMate 77T | Neoadjuvant+adjuvant  , phase III | Cycle: 4(Q3W) +12(Q4W)  Nivo+CTx→R→Nivo | IIA(>4cm)-IIIB(N2) (AJCC v8) | 35.4% vs. 12.1% | 25.3% vs. 4.7% | NA | HR, 0.58  (NR vs. 18.4 m) | NA | 37.1% |
| KEYNOTE-671 | Neoadjuvant+adjuvant  , phase III | Cycle: 4+13 (Q3W)  Pembro+CTx→R→Pembro | II-IIIB(N<3)(AJCC v8) | 30.2% vs. 11% | 18.1% vs. 4.0% | 62.4% vs. 40.6% | HR, 0.58  (NR vs. 17.0m) | HR, 0.72  NR | 40.4% |
| Neotorch | Neoadjuvant+adjuvant  , phase III | Cycle: 3+1+13(Q3W)  Toripalimab+CTx→R→Tori | II-III (AJCC v8) | 48.5% vs. 8.4% | 24.8% vs. 1.0% | 64.7% vs. 38.7% | HR, 0.40  (NR vs. 15.5m) | HR, 0.62  NR | 43.6% |
| AEGEAN | Neoadjuvant+adjuvant  , phase III | Cycle: 4(Q3W) +12(Q4W)  Durva+CTx→R→Durva | IIA-IIIB(N<3)(AJCC v8) | 33.3% vs. 12.3% | 17.2% vs. 4.3% | 63.3% vs. 52.4% | HR, 0.68  (NR vs. 25.9 m) | NA | 26.3% |

Abbreviation: Nivo: nivolumab; Pembro: Pembrolizumab; Durva: durvalumab; Tori: Toripalimab; NA:Not found; NR: Not reached; pCR: Pathological complete response; MPR: Major pathological response; AJCC: American Joint Committee on Cancer.
