# Supplementary material for: Prognostic Impact of Adjuvant Immunotherapy in Patients With Resectable NSCLC After Neoadjuvant Chemoimmunotherapy: A Brief Report
Source: JTO Clin Res Rep. 2024 Nov 12;6(1):100763. doi: 10.1016/j.jtocrr.2024.100763 (PMC11699361; doi:10.1016/j.jtocrr.2024.100763)
Supplement: Supplementary Table 2 [file mmc6.docx]

**Supplementary table 2.** Baseline comparison of adjuvant immunotherapy with and without in the entire cohort

|  | All patients | Adjuvant immunotherapy | Observation | P |
| --- | --- | --- | --- | --- |
| No. of patients | 438 | 176 | 262 |  |
| Age: |  |  |  | 0.028 |
| <65 | 232 (53.0%) | 105 (59.7%) | 127 (48.5%) |  |
| ≥65 | 206 (47.0%) | 71 (40.3%) | 135 (51.5%) |  |
| Gender: |  |  |  | 0.777 |
| Female | 39 (8.90%) | 17 (9.66%) | 22 (8.40%) |  |
| Male | 399 (91.1%) | 159 (90.3%) | 240 (91.6%) |  |
| Smoking history: |  |  |  | 0.488 |
| Never smoked | 157 (35.8%) | 67 (38.1%) | 90 (34.4%) |  |
| Current or Former smoker | 281 (64.2%) | 109 (61.9%) | 172 (65.6%) |  |
| PD-L1: |  |  |  | 0.264 |
| 0% | 203 (46.3%) | 86 (48.9%) | 117 (44.7%) |  |
| 1-49% | 96 (21.9%) | 33 (18.8%) | 63 (24.0%) |  |
| ≥50% | 58 (13.2%) | 28 (15.9%) | 30 (11.5%) |  |
| NA | 81 (18.5%) | 29 (16.5%) | 52 (19.8%) |  |
| Neoadjuvant cycles: |  |  |  | 0.270 |
| 2-3 | 350 (79.9%) | 134 (76.1%) | 216 (82.4%) |  |
| 4 | 73 (16.7%) | 35 (19.9%) | 38 (14.5%) |  |
| >4 | 15 (3.42%) | 7 (3.98%) | 8 (3.05%) |  |
| Histological type: |  |  |  | 0.569 |
| Non-sqcc | 150 (34.2%) | 57 (32.4%) | 93 (35.5%) |  |
| sqcc | 288 (65.8%) | 119 (67.6%) | 169 (64.5%) |  |
| Pathologic response (PT): |  |  |  | 0.123 |
| Non-MPR | 195 (44.5%) | 70 (60.8%) | 125 (47.7%) |  |
| MPR | 243(55.5%) | 106 (60.2%) | 137 (52.3%) |  |
| Node stage: |  |  |  | 0.490 |
| N0 | 278 (63.5%) | 117 (66.5%) | 161 (61.5%) |  |
| N1 | 64 (14.6%) | 22 (12.5%) | 42 (16.0%) |  |
| N2 | 96 (21.9%) | 37 (21.0%) | 59 (22.5%) |  |

Abbreviation: PD-L1, programmed cell death-Ligand 1; Sqcc, squamous cell carcinoma; MPR, major pathologic response; PT, primary tumor.
