# Supplementary material for: Prognostic Impact of Adjuvant Immunotherapy in Patients With Resectable NSCLC After Neoadjuvant Chemoimmunotherapy: A Brief Report
Source: JTO Clin Res Rep. 2024 Nov 12;6(1):100763. doi: 10.1016/j.jtocrr.2024.100763 (PMC11699361; doi:10.1016/j.jtocrr.2024.100763)
Supplement: Supplementary Table 3 [file mmc7.docx]

|  | Patients with ≥6 Cycles of Adjuvant Immunotherapy (N=122) | Patients with <6 Cycles of Adjuvant Immunotherapy (N=54) |
| --- | --- | --- |
|  | *no. (%)* | |
| Grade 3-5 | 13 (10.7) | 3 (5.6) |
| Serious | 5 (4.1) | 1 (1.9) |
| Led to death | 0 | 0 |
| Treatment-related adverse events of grade 3-5 |  |  |
| Rash | 3(2.5) | 1 (1.9) |
| Decreased neutrophil count | 3 (2.5) | 1 (1.9) |
| Anemia | 2 (1.6) | 1 (1.9) |
| Hypothyroidism | 2 (1.6) | 0 |
| Fatigue | 2 (1.6) | 0 |
| Pruritus | 1 (0.8) | 0 |

**Supplementary table 3. Treatment-Related Adverse Events that Occurred During the Adjuvant Treatment Phase**
